# Identification of New Rat Bone Marrow-Derived Population of Very Small Stem Cell with Oct-4A and Nanog Expression by Flow Cytometric Platforms

**DOI:** 10.1155/2016/5069857

**Published:** 2015-11-08

**Authors:** Anna Labedz-Maslowska, Elzbieta Kamycka, Sylwia Bobis-Wozowicz, Zbigniew Madeja, Ewa K. Zuba-Surma

**Affiliations:** Department of Cell Biology, Faculty of Biochemistry, Biophysics and Biotechnology, Jagiellonian University, 30-387 Krakow, Poland

## Abstract

Very small embryonic-like stem cells (VSELs) represent a unique rare population of adult stem cells (SCs) sharing several structural, genetic, biochemical, and functional properties with embryonic SCs and have been identified in several adult murine and human tissues. However, rat bone marrow- (BM-) derived SCs closely resembling murine or human VSELs have not been described. Thus, we employed multi-instrumental flow cytometric approach including classical and imaging cytometry and we established that newly identified population of nonhematopoietic cells expressing CD106 (VCAM-I) antigen contains SCs with very small size, expressing markers of pluripotency (Oct-4A and Nanog) on both mRNA and protein levels that indicate VSEL population. Based on our experience in both murine and human VSEL isolation procedures by fluorescence-activated cell sorting (FACS), we also optimized sorting protocol for separation of CD45^−^/Lin^−^/CD106^+^ rat BM-derived VSELs from wild type and eGFP-expressing rats, which are often used as donor animals for cell transplantations in regenerative studies *in vivo*. Thus, this is a first study identifying multiantigenic phenotype and providing sorting protocols for isolation VSELs from rat BM tissue for further examining of their functional properties *in vitro* as well as regenerative capacity in distinct *in vivo* rat models of tissue injury.

## 1. Introduction

Flow cytometric platforms have been well established as valuable tools for identification and isolation of several cell populations based on their multiantigenic profile [1–4]. Based on advanced modified and optimized FACS protocols, we have identified and sorted new fractions of rare stem cells (SCs) including very small embryonic-like stem cells (VSELs) that reside predominantly in bone marrow (BM) but also in other tissues such fetal liver, umbilical cord blood (UCB), and multiple adult specimens harvested from various organs and tissues [2, 3, 5]. The major impact of our experience in this subject was the implementation of challenging methods for purification of such unique rare fractions of SCs based on their multiantigenic profile by modern flow cytometric platforms.

Recently, numerous reports have shown that adult murine as well as human specimens such as BM, peripheral blood (PB), solid organs, and UCB may contain primitive stem cell fractions with multi- and pluripotent characteristics. Such SCs populations include unrestricted somatic stromal cells (USSCs) [6], multilineage-differentiating stress-enduring (Muse) cells [7, 8], marrow-isolated adult multilineage inducible cells (MIAMI) [9], multipotent adult progenitor cells (MAPCs) [10], multipotent adult stem cells (MASCs) [11], and a population of VSELs [12–14].

VSELs represent a unique rare population of adult SCs sharing several structural, genetic, biochemical, and functional properties with embryonic SCs and have been identified in several adult murine and human tissues including ovaries and testes [15–22]. Murine VSELs defined representing small-sized cells expressing Sca-1 antigen but not expressing CD45 and hematopoietic lineages markers (FSC^low^/SSC^low^/CD45^−^/Lin^−^/Sca-1^+^) have been initially identified in murine BM and subsequently found in several other adult murine organs as rare population of SCs [23–25]. Genetic analysis such as real-time RT-PCR in sorted murine FSC^low^/SSC^low^/CD45^−^/Lin^−^/Sca-1^+^ cells has showed the increased levels of mRNA for embryonic stem cells markers such as SSEA-1, Oct-4, Nanog, and Rex-1 (Rexo1) that was also confirmed on protein level using immunofluorescent staining and ImageStream system imaging (ISS) [23, 26]. Importantly, detailed molecular and genetic analysis of these cells reveled their (1) hypomethylated promoters for Oct-4 and Nanog transcription factors and (2) unique epigenetic status including hypomethylation of growth-repressive H19 gene along with hypermethylation of growth-promoting Igf-2 gene that leads to in inhibition of proliferation of these cells and limits their tumorigenic and blastocyst complementation capacity [27]. Importantly, the presence of VSELs in several other murine and human tissues including ovaries and testes has been also confirmed by other investigators [17–19, 21, 22, 28–30].

Human UCB- and PB-derived VSELs are phenotypically similar to those described in adult murine BM and may be also identified within nonhematopoietic compartments (CD45^−^/Lin^−^) of such specimens, especially among small-sized objects (FSC^low^/SSC^low^). Human VSELs are also very small in diameter and are smaller than red blood cells (RBCs), which is a unique feature for these stem cells along all investigated species. The population of Oct-4-, Nanog-, and SSEA-4-expressing VSELs in humans is enriched among CD45^−^/Lin^−^ fraction carrying CD133/1 (AC133), CD34, or partially CXCR4 [3, 4, 14]. Although the human VSELs have been initially characterized as cells expressing CXCR4 receptor, we further established that the fraction enriched in Oct-4, SSEA-4 expressing cells that possess very small size and high N/C ration, may be predominantly found in CD45^−^/Lin^−^/CD133^+^ population of UCB-derived cells [3, 31]. Such cell expressed early embryonic transcription factors as Oct-4 and Nanog, at both mRNA and protein levels as confirmed by quantitative RT-PCR and imaging cytometry, respectively [31]. Since then, we consider the CD45^−^/Lin^−^/CD133^+^ population as mostly enriched in VSELs. Importantly, cytometric characteristics of UCB-derived SCs revealed normal diploid (2n) content of DNA in both VSELs and HSCs fractions in the G0/G1 phase of the cell cycle [32].

Distinct “positive markers” have been identified for VSELs isolated from different species. In our previous studies, we have identified only limited number of such selection markers present on VSEL surface including Sca-1 antigen in mice and CD34 or CD133 in humans [32]. These findings indicate that the expression of these markers is species-related and there is no VSEL-specific surface antigen identified for all species up to today. Moreover, Sca-1 antigen representing murine VSELs selection marker is not present on human or rat cell, while CD34 antigen commonly present on human stem and progenitor cells has been rarely identified on murine or rat stem cells. Importantly, the positive selection markers for VSELs have not been identified within MSC-specific markers such as CD29 and CD105 [1, 7]. However, our long term observations of murine and human VSELs in* in vitro* culture strongly suggest their high adhesive properties. Such functional properties correspond to the expression of several adhesion molecules and receptors including CD54, CD106, and members of *α*-integrin family (e.g., CD49f) that were detected on murine VSEL surface by our group and also by Professor Ratajczak and his colleagues, but these important data are still unpublished. However these findings may suggest that the adhesion molecules and receptors may be group harboring selection markers for VSEL isolation that was considered in this study.

Although both murine and human VSELs have been well characterized on phenotypical and genetic levels, such primitive SCs in other mammals including rats have been poorly investigated. The first study questing for VSELs counterpart in rat BM was performed by Wu and colleagues who described a population of CD45^−^/Lin^−^ (Ter-119^−^, CD11b^−^, and Gr-1^−^)/SSEA-1^+^ cells in this tissue. Although the Sca-1 antigen has been identified specifically on murine, but not rat or human cells, the authors reported existence of such rat SSEA-1^+^ VSELs expressing Sca-1 and also PSC transcriptional factors such as Oct-4, Nanog, Rex-1, Sox-2, and Fgf-4 in murine bone marrow [33].

Thus, the aim of our study was to identify and purify enriched population of rat VSELs (rVSELs) derived from adult rat BM based on phenotypic and antigenic similarity to murine VSELs and selected antigen expression (CD54 and CD106) with using only specifically anti-rat reagents and by employing both classical and imaging flow cytometry (ImageStream X system, ISS-X). Moreover, the goal was to optimize flow cytometric sorting strategies for purification of rVSELs derived from wild type (WT) and eGFP-expressing transgenic Wistar rats that may be further employed in* in vitro* and preclinical studies identifying their functional potential.

## 2. Materials and Methods

### 2.1. Animals

Experimental procedures involving animal material were performed in accordance with the national and European legislation following approval by the First Local Ethical Committee on Animal Testing at the Jagiellonian University in Krakow (approval number: 56/2009). Two strains of Wistar rats, (1) WT (Crl: WT) and (2) transgenic eGFP-expressing rats (Wistar-TgN(CAG-GFP)184Ys), were purchased from National Bio Resource Project for the Rat in Japan, Kyoto University, and supplied by the experimental animal facility from Nencki Institute of Experimental Biology, Polish Academy of Sciences, Warsaw, Poland.

### 2.2. BM Cells Preparation

BM tissue was isolated from adult (8–12-week-old) WT and transgenic eGFP-expressing Wistar rats by flashing of cavities of tibias and femurs. BM cell suspensions were collected and filtered through a 70-*μ*m strainer (BD Bioscience). Total nucleated cells (TNCs) were obtained following lysis of red blood cells (RBCs) with 1x BD Pharm Lyse Buffer (BD Pharmingen) for 10 minutes. TNCs were subsequently washed with phosphate buffered saline (DPBS; w/o Ca^2+^, Mg^2+^; Life Technologies) and resuspended in DMEM-based staining medium (Sigma-Aldrich, St. Louis, MO) containing 2% of fetal bovine serum (FBS) (Lonza, Basel, Switzerland) for further analyses.

### 2.3. Staining for Flow Cytometric Analysis and FACS Sorting

TNCs harvested from both Wistar strains were immunolabeled with the following monoclonal antibodies: Alexa Fluor 647- or PE-Cy7-conjugated anti-CD45 (clone: OX-1; BioLegend), Alexa Fluor 647- or FITC-conjugated antihematopoietic lineages markers cocktail—“Lin” markers, including anti-TCR*αβ* (clone: R73), anti-CD3 (clone: 1F4), anti-CD11b/c (clone: OX-42), and anti-CD45RA (clone: OX-33); BD Pharmingen and BioLegend, PE-conjugated anti-CD54 (clone: 1A29; BD Pharmingen), and PE- or biotin-conjugated anti-CD106 (PE, clone: MR-106; BioLegend). Streptavidin with PerCp-Cy5.5 (BD Pharmingen) was added to the samples to visualize the binding of biotin-conjugated anti-CD106 antibody. All antibodies were used according to manufacturer's protocols and staining was performed for 30 min at 4°C. Cells were subsequently washed and resuspended in DMEM with 2% FBS for further flow cytometric analysis (LSR II; Becton Dickinson) or sorting procedure (MoFlo XDP; BeckmanCoulter).

Rat BM-derived VSEL population was considered to be enriched in FSC^low^/SSC^low^/CD45^−^/Lin^−^ cells expressing CD54 or/and CD106 antigens.

To compare the status of the sorted rVSELs with CD45^−^/Lin^−^/SSEA-1^+^ population identified by Wu et al. [33], TNC fraction was stained for CD45 and Lin markers (as described above) and additionally with directly conjugated anti-mouse/human SSEA-1 antibody (PE, clone: MC-480, BioLegend). We used directly conjugated anti-SSEA-1 antibody to prevent nonspecific background staining crucial to be avoided in sorting of rare cell populations. All used antibodies are described in Table 1.

### 2.4. Staining for ImageStream X System (ISS-X) Analysis

For rVSELs identification by ImageStream X system (Amnis Corp.), TNCs derived for WT Wistar rats were prepared according to the protocol described above. Based on the detection channels available for the ISS-X, the following directly conjugated monoclonal antibodies were used for identification of small CD45^−^/Lin^−^/CD106^+^ and/or CD45^−^/Lin^−^/CD54^+^ cells: (i) anti-CD45 (Alexa Fluor 647, clone: OX-1), (ii) FITC-conjugated Lin markers (including anti-TCR*αβ* (clone: R73), anti-CD3 (clone: 1F4), anti-CD11b/c (clone: OX-42), anti-CD45RA (clone: OX-33)) (BD Pharmingen), and (iii) CD106 (PE, clone: MR-106; BioLegend) or (iv) CD54 (PE, clone: 1A29) (BD Pharmingen). Staining was performed according to manufacturers' protocols for 30 min at 4°C. Samples were subsequently washed and fixed with 4% of paraformaldehyde solution (Sigma) for 20 min (RT). Fixed cells were resuspended in saline (DPBS; w/o Ca^2+^, Mg^2+^; Life Technologies) in concentration of 2 × 10^6^/mL for further analysis with ImageStream X system (Amnis Corp.); Hoechst 33342 (Hoe; 2 *μ*M) was added for 10 min prior to analysis to visualize nuclei.

In order to analyze intranuclear expression of pluripotent markers such as Oct-4A and Nanog and to further identify Oct-4A^+^ and/or Nanog^+^ rat BM-derived VSELs, freshly isolated TNCs were initially fixed with 4% paraformaldehyde (Sigma) for 20 min (RT) and subsequently permeabilized with 0.1% Triton X-100 solution (Sigma) for 10 min (RT). Cells were further washed and stained either with (i) primary anti-Oct-3/4 antibody (rabbit polyclonal IgG, Santa Cruz Biotechnology, sc-9081, CA, USA, 1 : 100) that specifically stains Oct-4A isoform identifying PSCs or (ii) primary anti-Nanog antibody (rabbit polyclonal IgG, Santa Cruz Biotechnology, sc-33760, 1 : 100) for 2 h at 37°C. Secondary goat anti-rabbit IgG antibody conjugated with Alexa Fluor 488 or Alexa Fluor 647 (Invitrogen, Molecular Probes, Carlsbad, Ca, USA, 1 : 200) was added for 2 h (37°C). Cells were washed following the staining for Oct-4A and Nanog and subsequently incubated with directly conjugated antibodies against (i) CD106-PE, CD45-PE-Cy7, or FITC-conjugated Lin markers (including TCR*αβ*, CD3, CD11b, and CD45RA) for 30 min at 4°C. Cells were further washed and resuspended in DPBS (w/o Ca^2+^, Mg^2+^; Life Technologies). Nuclei were stained with Hoechst 33342 (Hoe) directly before analysis and samples were further analyzed with ImageStream X system (Amnis Corporation, Seattle, WA, USA). Analyses of morphological features of selected BM-derived populations were performed based on the collected images with IDEAS analytical software (Amnis Corporation, Seattle, WA, USA).

### 2.5. Gene Expression Analysis by Real-Time RT-PCR

Total RNA from sorted subpopulations of CD45^−^/Lin^−^/CD106^+^/CD54^+^, CD45^−^/Lin^−^/CD106^+^, and CD45^−^/Lin^−^/SSEA-1^+^ cells as well as from their hematopoietic counterparts (CD45^+^/Lin^−^/CD106^+^/CD54^+^ and CD45^+^/Lin^−^/CD106^+^) was isolated using the RNeasy Micro Kit (Qiagen, Valencia, CA). mRNA was further reversely transcribed into cDNA with TaqMan Reverse Transcription Reagents (Applied Biosystems, Foster City, CA, USA) and the reactions were performed under the following conditions: 1 cycle at 25°C for 10 min, 1 cycle at 48°C for 30 min, and finally 1 cycle at 95°C for 5 min. mRNA harvested from unfractionated TNCs was used as control (called “input”). 50 ng of total RNA was used for each reverse transcription reaction.

Detection of expression of the following rat genes *β*-actin, Oct-4, Nanog, and Rexo1 was performed by real-time RT-PCR using an ABI PRISM 7000 sequence detection system (Applied Biosystems). A 25-*μ*L reaction mixture contained 12.5 *μ*L SYBR Green PCR Master Mix (Applied Biosystems), cDNA template (2,5 ng), and both forward (1 *μ*M) and reverse (1 *μ*M) primers (Tib MolBiol, Poznan, Poland). The following sequences of primers were used: *β*-actin: (F) 5′- TGA CCC AGA TCA TGT TTG AGA -3′, (R) 5′- CAA GGT CCA GAC GCA GGA T-3′; Oct-4: (F) 5′- CCC AGC GCC GTG AAG TTG GA -3′, (R) 5′- AGA ACG CCC AGG GTG AGC CC -3′; Nanog: (F) 5′- CCC TTG CCG TTG GGC TGA CA -3′, (R) 5′- AAG GCG GAG GAG AGG CAG TCT -3′; Rexo-1 (F) 5′- GCT CCG GCG GAA TCG AGT GG -3′, (R) 5′- GCA CGT GTT GCT TGG CGA CC -3′.

Real-time RT-PCR reactions were performed under the following conditions: 1 cycle at 50°C for 2 min, 1 cycle at 95°C for 10 min, followed by 40 cycles at 95°C for 15 s, and 60°C for 1 min. Relative quantification of Oct-4, Nanog, and Rexo1 mRNA expression was calculated using the comparative Ct method. The relative quantitative value of the target, normalized to an endogenous control (*β*-actin gene) and relative to a calibrator, was expressed as 2^−ΔΔCt^ (i.e., fold difference), where ΔCt = [Ct of target genes] − [Ct of endogenous control gene] and ΔΔCt = [ΔCt of samples for target genes] − [ΔCt of calibrator for target gene].

To avoid the possibility of amplifying contaminating DNA, the following steps were taken: (i) all primers were designed with an intron sequence inside the cDNA to be amplified; (ii) all reactions were performed with an appropriate negative controls (template-free controls); (iii) a uniform amplification of the products was rechecked by analyzing the melting curves of the amplified products (dissociation graphs).

### 2.6. Immunocytochemistry

To evaluate Oct-4A and Nanog expression in purified BM subpopulation, the limited number of sorted cells (10 × 10^3^) was seeded on poly-L-lysine (Sigma) coated glass-bottom plates (Willco-dish, Willco Wells B.V.) in medium DMEM with 2% FBS (Lonza) and was incubated overnight in standard culture conditions. The medium was subsequently removed and the attached cells were gently fixed with 4% paraformaldehyde (Sigma) for 20 min (RT) and subsequently permeabilized with 0.1% Triton X-100 solution (Sigma) for 10 min (RT). Cells were washed and stained either with (i) primary anti-Oct-3/4 antibody (rabbit polyclonal IgG, Santa Cruz Biotechnology, sc-9081, CA, USA, 1 : 100) that specifically stains Oct-4A isoform identifying PSCs or (ii) primary anti-Nanog antibody (rabbit polyclonal IgG, Santa Cruz Biotechnology, sc-33760, 1 : 100) for 16 h at 4°C. Secondary goat anti-rabbit IgG antibody conjugated with Alexa Fluor 488 or Alexa Fluor 546 (Invitrogen, Molecular Probes, Carlsbad, Ca, USA, 1 : 200) was added for 2 h (37°C). Cells were washed and nuclei were stained with 4′, 6-diamidino-2-phenylindole (DAPI, 2 *μ*M, Life Technologies, Molecular Probes) for 15 min (37°C) and plates were closed with VECTASHIELD Mounting Medium (Vector Laboratories, CA, USA) and cover slips. Sorted CD45^+^/Lin^−^/CD106^+^ subpopulation of BM cells that represents hematopoietic counterpart of purified CD45^+^/Lin^−^/CD106^+^ VSELs was also stained for Oct-4A and Nanog and was used as negative control in this setting. The labeled cells were subsequently analyzed with Leica DMI6000B (ver. AF7000) fluorescent microscope under total 945x magnification (Leica Microsystems GmbH, Germany).

### 2.7. Statistical Analysis

Data were expressed as the mean ± standard deviation (SD). A value of *P* < 0.05 was considered significant. All statistical analyses were performed using the Origin (ver. 5.0) statistical software (Microcal Software, Northampton, MA).

## 3. Results

### 3.1. Rat BM Harbors a Population of Very Small Nonhematopoietic SCs Expressing CD54 and CD106 Antigen

Our previously published data have indicated murine and human VSELs represent populations of nonhematopoietic stem cells with very small cells size which do not express markers of hematopoietic lineages such as CD45 and major hematopoietic lineage specific antigens [2, 3, 5]. Thus, in this study, we started our quest for rat BM-derived VSELs focusing on cells with corresponding characteristics by employing multiparameter flow cytometric platforms. We focused on CD45^−^/Lin^−^ cells with small size and low cytoplasmic complexity and volume, indicated as FSC^low^ and SSC^low^ objects, respectively. Our second step was focused on “positive selection” marker that would optimally define these unique SCs. We have previously reported only limited number of markers present on VSEL surface that may distinguish them from other cell types such as Sca-1 antigen in mice and CD34 or CD133 in humans [2, 3, 5]. Therefore, the expression of VSEL “positive selection” markers is distinct in different species and VSEL-specific surface antigen has not been identified. However, our long term observations of murine and human VSELs in* in vitro* culture indicate their high adhesive properties which correspond to the expression of several adhesion molecules and receptors including, for example, CD54, CD106, and CD49f (unpublished data). Thus, we focused on two molecules involved in cell adhesion as potential markers for rVSELs including CD54 (ICAM-I) and CD106 (VCAM-I).

We initially isolated BM cells from adult WT Wistar rats and stained total nucleated cells obtained following RBCs lysis to identify presence of CD45^−^/Lin^−^/CD54^+^ or CD45^−^/Lin^−^/CD106^+^ cells by classical as well as imaging cytometry (ISS-X) that allows for distinguishing real rare cellular objects from artifacts.

**Figure 1:**
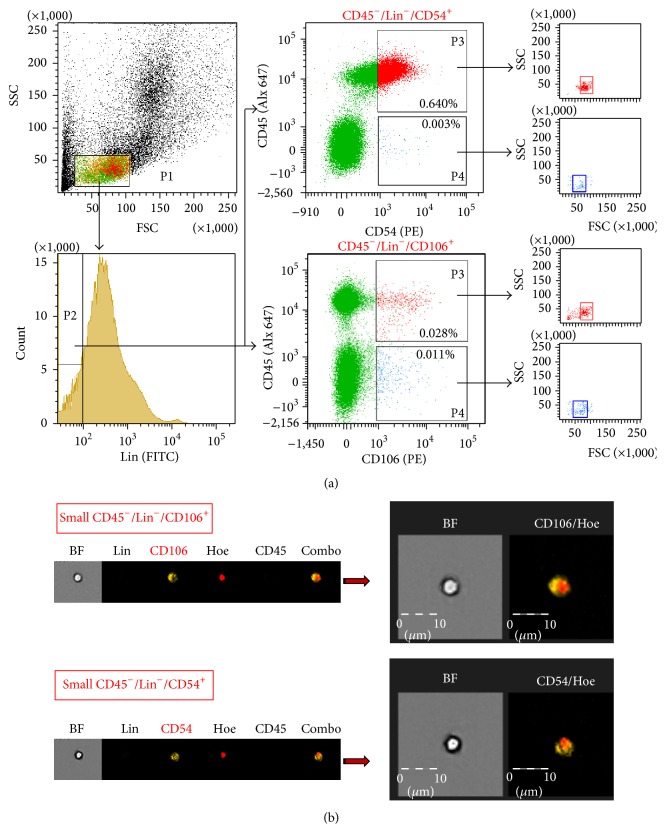
Expression of CD54 (ICAM-I) and CD106 (VCAM-I) on rat BM cells. (a) Gating strategy for identification of CD54^+^ and CD106^+^ populations by classical flow cytometry. Total nucleated cells (TNCs) derived from WT rat BM were stained for CD45 (PE-Cy7), hematopoietic lineages markers (Lin: TCR*αβ*, CD3, CD11b, CD45RA; FITC), and CD54 (PE) or CD106 (PE) and further analyzed by LSR II (Becton Dickinson). BM cells are visualized on dot-plot showing FSC versus SSC signals, which are related to the size and granularity/complexity of the cell, respectively. 2% of total 1 × 10^6^ of analyzed TNCs is only displayed in this dot-plot to visualize the population distribution. Objects from region P1 (lymphgate including FSC^low^/SSC^low^ objects) were further analyzed for Lin markers expression and only Lin^−^ events are included into region P2.* Upper, middle dot-plot* cells stained for CD54, derived from gate P2, and analyzed for CD54 versus CD45 expression. Two fractions of cells potentially enriched in stem/progenitor cells are gated: rHSCs, CD45^+^/Lin^−^/CD54^+^ (region P3), and rVSELs, CD45^−^/Lin^−^/CD54^+^ (region P4). Both populations are further “back-gated” on FSC versus SSC dot-plot to visualize cell size distribution.* Lower, middle dot-plot* cells stained for CD106, derived from gate P2, and analyzed for CD106 versus CD45 expression; rHSC, CD45^+^/Lin^−^/CD106^+^ (region P3), and rVSELs, CD45^−^/Lin^−^/CD106^+^ (region P4). Both populations are further “back-gated” on FSC versus SSC dot-plot to visualize cell size distribution. Percentages show content of each subpopulation among TNCs in representative sample. Total 1 × 10^6^ of TNCs was typically collected for each sample to identify the SC populations. (b) Representative images of nonhematopoietic cells expressing CD54 and CD106 by ImageStream X system (ISS-X). TNCs were stained for Lin markers (FITC, green), CD45 (Alexa Fluor 647, blue), CD54 (PE, orange), or CD106 (PE, orange). Cells were fixed and nuclei were stained with Hoechst 33342 prior to analysis by ISS. Extracellular expression of CD106 and CD54 is shown on combo as well as on magnified images (right panels). The scale bars indicate 10 *μ*m.

By using both flow cytometric platforms, we found that rat BM tissue contains both rare populations of very small nonhematopoietic (CD45^−^/Lin^−^) cells expressing CD54 and CD106 antigens (0.003 ± 0.001% and 0.011 ± 0.005%, resp.) (Figure 1). Since we expected rVSELs to be small size cells, we focused on gating events including those with low values of FSC and SSC parameters (Figure 1(a)). We found that especially CD45^−^/Lin^−^/CD106^+^ fraction exhibited morphology of very small cells in comparison with its hematopoietic counterpart (CD45^+^/Lin^−^/CD106^+^) when “back-gated” on FSC versus SSC dot-plot (Figure 1(a)). Moreover, we confirmed the existence of such small cellular objects within both identified populations of CD45^−^/Lin^−^/CD54^+^ or CD45^−^/Lin^−^/CD106^+^ cells by ImageStream system (Figure 1(b)).

### 3.2. Rat BM-Derived CD45^−^/Lin^−^/CD106^+^ Population Contains Developmentally Early rVSELs Expressing Oct-4 and Nanog Pluripotency Markers

**Figure 2:**
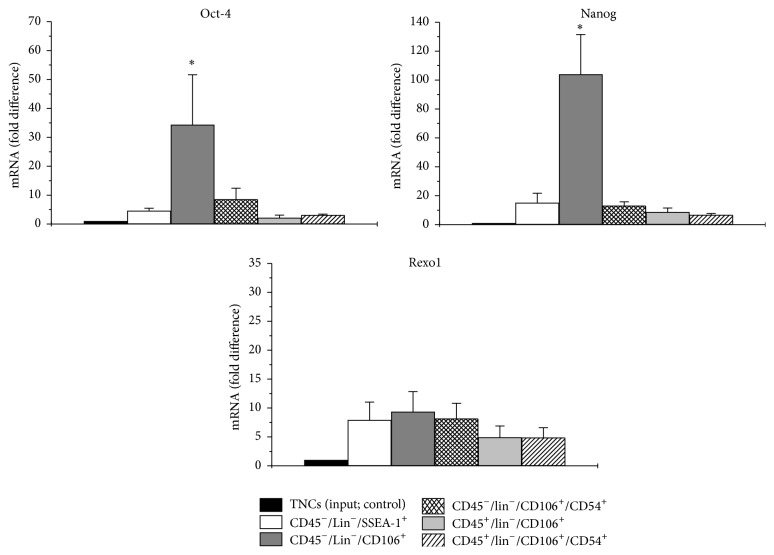
Expression of mRNA for Oct-4, Nanog, and Rexo-1 by real-time RT-PCR in sorted fractions of BM cells. Indicated populations of nonhematopoietic (CD45^−^/Lin^−^/CD106^+^, CD45^−^/Lin^−^/CD106^+^/CD54^+^, and CD45^−^/Lin^−^/SSEA-1^+^) and hematopoietic (CD45^+^/Lin^−^/CD106^+^, CD45^+^/Lin^−^/CD106^+^/CD54^+^) cells were sorted with MoFlo XDP. The graphs show the fold difference in concentration of mRNA for Oct-4, Nanog, and Rexo-1 in sorted fractions when compared to unfractionated TNCs (shown as 1). Results are presented as mean ± SD. Statistically significant differences (*P* < 0.05) are shown when compared with TNCs. Analysis was performed three times with samples prepared from three independent sorts.

Thus, by employing two staining and gating strategies on MoFlo XDP cell sorter, we focused on two subpopulations expected to potentially contain rVSELs: (1) CD45^−^/Lin^−^/CD106^+^ and (2) CD45^−^/Lin^−^/CD106^+^/CD54^+^. We further evaluated the expression of selected VSEL-related genes (Oct-4, Nanog, and Rexo1) whose expression we consider as indicator for the potential presence of rVSELs in such sorted fractions (Figure 2). Additionally to hematopoietic counterparts (CD45^+^/Lin^−^/CD106^+^/CD54^+^ and CD45^+^/Lin^−^/CD106^+^ cells), we also purified nonhematopoietic fraction (CD45^−^/Lin^−^) of rat BM cells expressing SSEA-1 which has been recently reported as rat VSEL population by Wu et al. [33]. We planned to pursue this fraction further in our experiments if enrichment in mRNA for VSEL-related genes is found within these cells. We found significantly greater concentration in mRNA for both vast transcription factors regulating cell pluripotency (Oct-4A and Nanog) in purified CD45^−^/Lin^−^/CD106^+^ population of rat BM cells when compared to unfractionated BM cells (TNCs; input cells) and also to CD45^−^/Lin^−^/SSEA-1^+^ fraction (Figure 2, Table 2). However, opposite to our initial expectations, the expression of Oct-4 and Nanog in highly purified subfraction of CD45^−^/Lin^−^/CD106^+^/CD54^+^ cells was lower than in CD45^−^/Lin^−^/CD106^+^ population (Figure 2, Table 2). These results indicated that CD54 was not a selection marker for Oct-4-expressing rVSELs and other markers need to be found to be coexpressed with CD106 and to enrich for rVSELs during sorting procedure.

We could also notice some upregulation in mRNA expression for Oct-4 and Nanog in CD45^−^/Lin^−^/SSEA-1^+^ cells when compared to unpurified BM cells, but it was rather negligible and significantly lower when compared with purified CD45^−^/Lin^−^/CD106^+^ population (Figure 2, Table 2). The level of mRNA expression for Rexo1 that also represents transcription factor expressed in pluripotent cells occurred to be the least discriminative for the tested populations and was fairly enriched in all three sorted nonhematopoietic populations (Figure 2, Table 2). Thus, the results indicate that the expression of CD106, but not CD54 and SSEA-1 antigens, defines developmentally early population of SCs in rat bone marrow.

**Figure 3:**
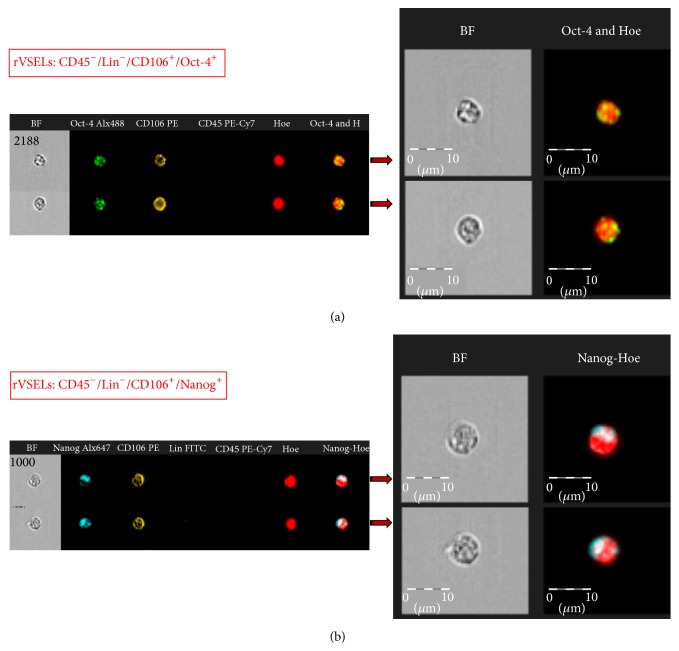
Expression of Oct-4A and Nanog in selected BM populations on protein level. Representative images of Oct-4A- and Nanog-expressing CD45^−^/Lin^−^/CD106^+^ cells by ImageStream X system. TNCs were stained for (1) Oct-4A (Alexa Fluor 488, green), CD45 (PE-Cy7, magenta), and CD106 (PE, orange) (a) and (2) Nanog (Alexa Fluor 647, blue), Lin markers (FITC, green), CD45 (PE-Cy7, magenta), and CD106 (PE, orange) (b). Cell were stained with Hoechst 33342 to visualize nuclei and analyzed by ISS to detect intranuclear expression of Oct-4A (a) and Nanog (b) as shown in magnified, combined images. The scale bars indicate 10 *μ*m.

**Figure 4:**
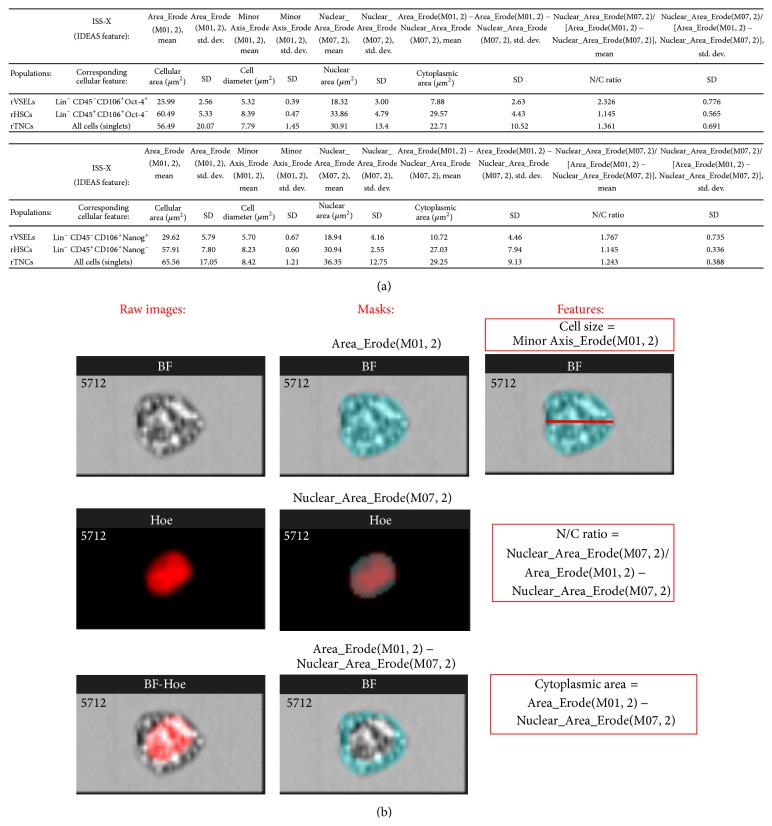
Quantitative analysis of morphological features of selected rat BM populations by ImageStream X system. (a) Quantitative analysis of cell size, nuclear to cytoplasmic (N/C) ratio, and cytoplasmic area of rVSELs when compared to other cell types. Analysis was performed based on the collected images of following rat BM populations: (i) rVSELs: CD45^−^/Lin^−^/CD106^+^ cells expressing Oct-4A or Nanog (upper and lower table, resp.); (ii) rHSCs: CD45^+^/Lin^−^/CD106^+^ cells with no expression of Oct-4A and Nanog, and (iii) TNCs (gated as singlets during the analysis). Analyses were performed with IDEAS software (Amnis Corp.). Data are shown as average values of each indicated feature within the cell population (mean ± SD). (b) Analytical approach for masking and feature calculation by IDEAS. Images show brightfield (BF), nuclear (Hoe), and combined (BF-Hoe) images of one example rat BM cell as well as exact masks (cyan) and features (formulas based on the defined masks) that were optimized and used for the analysis shown in (a). Cell size was expressed as minor cell axis computed within the mask covering BF image, while cytoplasmic area and N/C ratio were calculated based on the areas of BF and nuclear images (as indicated in red boxes). All masks and features including their nomenclature represent standard parameters provided by IDEAS software (Amnis Corp.)

**Figure 5:**
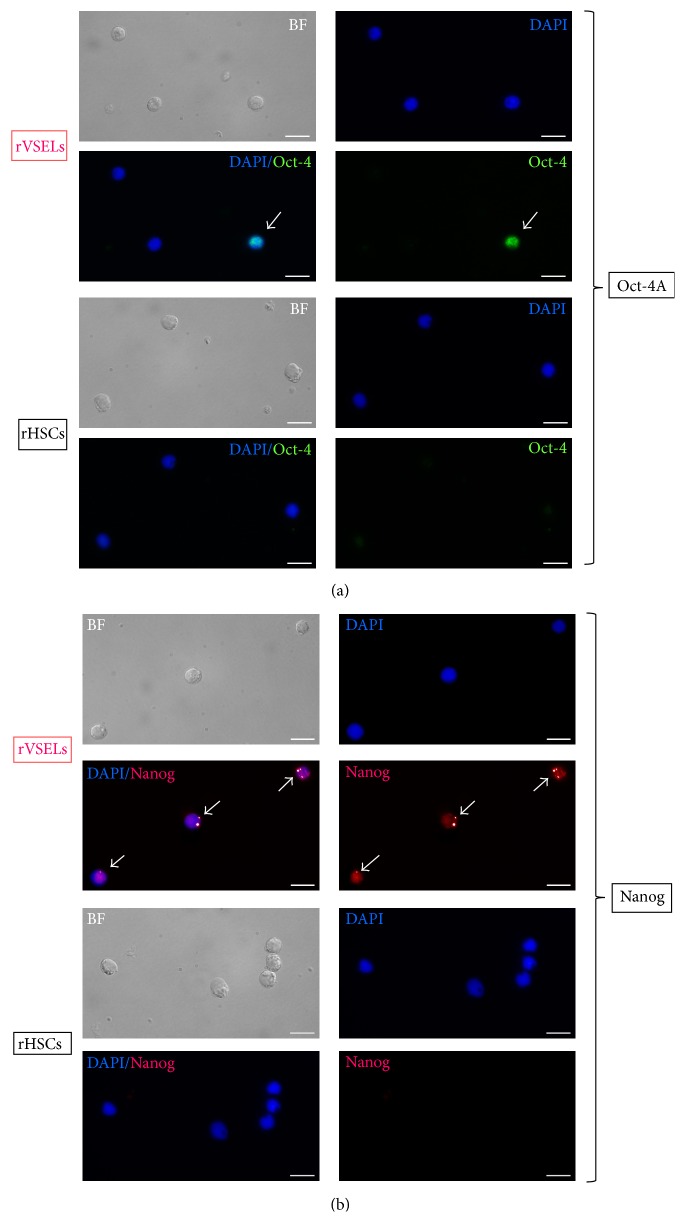
Expression of Oct-4A and Nanog in sorted fractions of rVSELs (CD45^−^/Lin^−^/CD106^+^) and rHSCs (CD45^+^/Lin^−^/CD106^+^) by immunocytochemistry. Both fractions were sorted with MoFlo XDP cell sorter (Beckman Coulter) and further stained for Oct-4A (Alexa Fluor 488, green) (a) or Nanog (Alexa Fluor 546, red) (b) and analyzed with Leica DMI6000B (ver. AF7000) fluorescent microscope. Nuclei are stained with DAPI. Intranuclear staining for both transcription factors is visualized on combo images. The scale bars indicate 10 *μ*m.

Since our data from gene expression analysis indicated that the fraction of CD45^−^/Lin^−^/CD106^+^ cells may be enriched in primitive SCs, we evaluated expression of Oct-4 and Nanog on protein level within (i) CD45^−^/Lin^−^/CD106^+^ subpopulation gated on whole BM cells by ImageStream X system and we calculated morphological features of these cells (Figures 3 and 4) as well as in (ii) FACS-purified CD45^−^/Lin^−^/CD106^+^ fraction by immunocytochemistry (Figure 5). Importantly, in terms of identification of expression of Oct-4, we employed exclusively antibodies that bind Oct-4A isoform related to stem cell pluripotency to focus entirely on SCs with such characteristics (Santa Cruz Biotechnology, sc-9081, CA, USA).

Both imaging platforms confirmed the existence of subpopulation of stem cells expressing Oct-4A and Nanog among CD45^−^/Lin^−^/CD106^+^ fraction of rat BM cells (Figures 3 and 5). On the other hand, we did not observe any cells expressing Oct-4 or Nanog among sorted hematopoietic counterparts (Figure 5). We also directly measured several morphological features of Oct-4 expressing CD45^−^/Lin^−^/CD106^+^ rVSELs when compared to other cells including diameter of individual cells (cell size) as well as N/C ratio and cytoplasmic area that confirmed primitive stem cell phenotype of rVSELs (Figure 4). We found that CD45^−^/Lin^−^/CD106^+^/Oct-4^+^ rVSELs possess the smallest size, the highest N/C ratio, and the smallest cytoplasmic area when compared to CD45^−^/Lin^−^/CD106^+^ rHSCs and TNC population (Figure 4).

Importantly, only subfraction of CD45^−^/Lin^−^/CD106^+^ cells stained positively for Oct-4A (Figure 5) indicating the need for further purification of these cells leading eventually to further enrichment in pluripotent rVSELs.

### 3.3. Optimized Protocol for Isolation of Rat BM-Derived rVSELs-Technical Hints

**Figure 6:**
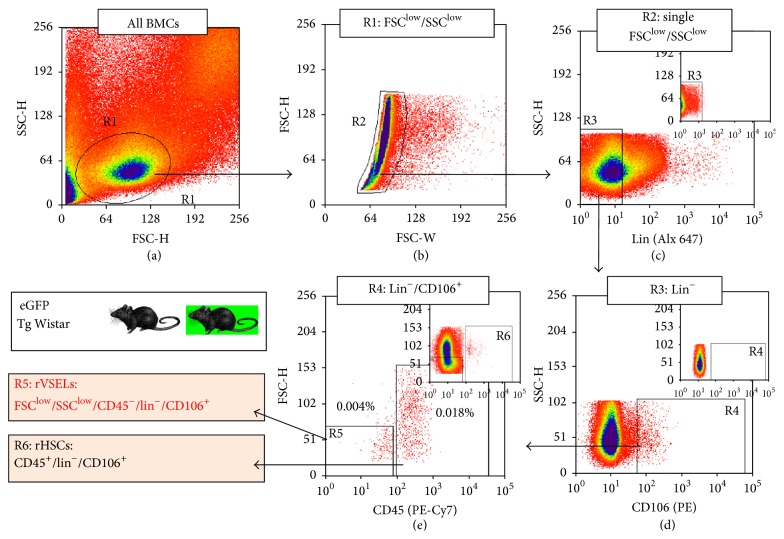
Optimized protocol for isolation of rVSELs from WT and eGFP-expressing rat BM tissue. Rat BM-derived VSELs were isolated from full population of BM cells stained for CD45 (PE-Cy7), Lin markers (TCR*αβ*, CD3, CD11b, and CD45RA; Alexa Fluor 647), and CD106 (PE) by MoFlo XDP cell sorter (Beckman Coulter). (a) Total nucleated cells (TNCs) are visualized on dot-plot showing FSC-H versus SSC-H. (b) Small agranular cells from gate R1 (with extension of lymphgate into low values of FSC; Figure 5(a)) are plotted on FSC-W versus FSC-H dot-plot to exclude doublets. (c) Single cells from gate R2 are subsequently analyzed for Lin markers expression. (d) Lin^−^ events included in region R3 are further plotted on dot-plot showing CD106 expression versus side scattered characteristics (SSC-H) of these cells. (e) CD106^+^ cells from gate R4 are eventually visualized on dot-plot based on their CD45 expression and FSC signal (FSC-H). Rat HSCs are identified as CD45^+^/Lin^−^/CD106^+^ (region R6), while rVSELs as FSC^low^/SSC^low^/CD45^−^/Lin^−^/CD106^+^ cells (region R5).

Since we established that CD45^−^/Lin^−^/CD106^+^ population of rat BM cells exhibit higher concentration of mRNA for pluripotent stem cell markers such as Oct-4 and Nanog which was also confirmed on protein level, we moved to fine-tuning the sorting protocol for such SCs that would be applicable for isolation of these cells from both WT and eGFP-expressing rat BM tissue (Figure 6). We focused on BM tissue derived not only from WT, but also from transgenic eGFP-expressing rats, since the potential donor eGFP+ rVSELs may be tracked following transplantation into WT animals, which would be important for further studies of their biological properties* in vivo*.

Thus, we employed the following directly conjugated antibodies prior to sorting procedures to avoid any nonspecific binding: (i) Alexa Flour 647/APC-conjugated antibodies against Lin markers (anti-TCR*αβ*, anti-CD3, anti-CD11b/c, and anti-CD45RA); (ii) PE-Cy7-conjugated antibody for CD45, and (iii) PE-conjugated antibody for CD106 (all antibodies listed in Table 1) that allow for compensation and sorting also eGFP-expressing cells (Figure 6).

In the first gating step, all events including lymphocytes and sublymphocyte fraction with FSC^low^/SSC^low^ parameters (enclosing small cell and debris) were included into sorting gate R1 (“*lymphgate extended into lower values of FSC*”; Figure 6(a)). In the next step of such gating strategy, doublets were excluded from sorting by employing gating on FSC-Width versus FSC-Height dot-plot (Figure 6(b)). Single cells from gate R2 were subsequently analyzed based on hematopoietic lineages (Lin) markers expression and Lin^−^ fraction derived from gate R3 (Figure 6(c)) was further visualized based on CD106 expression (Figure 6(d)). CD106^+^ cells were finally gated based on CD45 expression on CD45 versus FSC-H dot-plot which allowed us to distinguish two populations of (1) rat VSELs (FSC^low^/SSC^low^/CD45^−^/Lin^−^/CD106^+^) and (2) hematopoietic CD45^+^/Lin^−^/CD106^+^ cells consisting of smaller and larger subfractions (Figure 6(e)). Importantly, the established new protocol for FACS sorting of CD106-expressing very small cells from rat bone marrow allows for isolation of developmentally early SCs expressing Oct-4 and Nanog antigens from tissues of both WT and GFP animals.

## 4. Discussion

Recent evidence indicates that adult murine BM harbors a multitude of nonhematopoietic stem and progenitor cells in addition to well described HSCs [34, 35]. Such SC populations belonging to nonhematopoietic compartment of BM tissue include mesenchymal stem cells (MSC) [36], multipotent adult progenitor cells (MAPC) [10], marrow-isolated adult multilineage inducible cells (MIAMI) [9], multipotent adult stem cells (MASC) [11], and very small embryonic-like stem cells (VSEL) [31].

BM-derived murine VSELs have been isolated and characterized based on surface antigens as well as gene expression as a population of Sca-1^+^/Lin^−^/CD45^−^/Oct-4^+^/Nanog^+^ cells [23]. VSELs morphology and ultrastructure have been also described indicating their very small size (smaller than erythrocytes) and higher N/C ratio which supports their primitive nature [24]. Importantly, several groups have reported the presence of such SCs in other murine and human tissues including ovaries and testes [16–22, 28, 29].

In this study, we isolated and antigenically defined in adult rat BM a population of stem cells with developmentally early characteristics that we may define as FSC^low^/SSC^low^/CD45^−^/Lin^−^/CD106^+^ cells. Due to a very small size and the presence of other stem cell-related morphological features accompanied with expression of markers related to pluripotency including expression of Oct-4 and Nanog, we consider this population as rat counterpart of BM-derived murine VSELs. As such we called these cells rat VSELs (rVSELs).

In the first step, we tested and optimized two different protocols for sorting rVSELs by flow cytometry. The unique small size of VSELs requires optimized strategy for sorting that includes minimal threshold on instrument and extension the “lymphgate” into lowest values of FSC signal as described in several previous studies where VSELs were isolated [14, 25]. Based on the established protocols, we focused in our sorting gating strategy exclusively on small objects including lymphocytes and sublymphocyte fraction with FSC^low^/SSC^low^ parameters that may enclose very small stem cells. Such approach in isolation of VSEL from murine and human specimens has been previously employed and successfully used by several groups [3, 15, 17, 25, 29] and represents a crucial step for successful isolation of these rare cells.

In the next step, we selected potential markers for purification of rat VSELs including CD54 and CD106 representing vast surface antigen potentially expressed on subset on murine VSELs. Although CD54 (ICAM-1) antigen has been found to be expressed on activated endothelial cells, T and B cells, monocytes/macrophages, granulocytes, and dendritic cells [37], it may also be expressed on stem cells [38, 39]. Similarly, CD106 (VCAM-1) may be expressed not only on mature myeloid cells, splendid dendritic cells, or induced endothelial cells, but also on more primitive bone marrow stem/stromal cells [40]. CD106 represents a counterreceptor for VLA-4 (*α*_4_ *β*_1_ integrin) [41] and the interactions between these molecules may play an important role in binding of leukocytes to activated endothelial cells and leukocyte extravasation at inflammatory sites as well as in stem cell migration [41, 42].

By employing multi-instrumental flow cytometric analysis, quantitative RT-PCR, and immunochemistry, we established that FACS-sorted population of FSC^low^/SSC^low^/CD45^−^/Lin^−^/CD106^+^ rat VSELs expresses several markers of pluripotency including Oct-4, Nanog, and Rexo1. We established that isolation protocol described in this study may be more effective in isolation of developmentally early SCs when compared to FSC^low^/SSC^low^/CD45^−^/Lin^−^/CD54^+^ population or CD45^−^/SSEA-1^+^ cells described by Wu and colleagues [33]. Here, for the first time we report that highly purified adult rat BM-derived CD45^−^/Lin^−^/CD106^+^ cells may be enriched in fraction of SCs expressing markers of pluripotent cells such as Oct-4 and Nanog. Importantly, such CD45^−^/Lin^−^/CD106^+^ population remains still heterogeneous in terms of the expression of both markers indicating that further phenotypic characterization has to be performed. Interestingly, coexpression of CD54 does not indicate the population of rVSELs with greater Oct-4 and Nanog expression. Although single small cellular objects were found within CD45^−^/Lin^−^/CD54^+^ population by ISS, we lost enrichment in early Oct-4- and Nanog-expressing rVSELs in CD45^−^/Lin^−^/CD106^+^/CD54^+^ fraction. The data indicates that other surface markers will be required in the future to enrich purification of the Oct-4- and Nanog-expressing rVSELs.

Importantly, since some cellular debris may be also sorted during rVSELs purification due to the very small size expected from stem cells, the imaging cytometry (ImageStream X; ISS-X) was also employed in our study to confirm that isolated rVSELs represent cellular nucleated objects. The ISS technology allows for statistical analysis of a variety of cellular parameters and for visualization of cells in suspension during flow analysis via high-resolution brightfield, darkfield, and fluorescence images collected with the high resolution [43–45]. Importantly, we have previously employed imaging cytometry for identification and characterization of VSELs from other specimens proving this technology as an optimal tool distinguishing real cellular objects such as small SCs from debris and artifacts present in different specimens [24, 25].

Interestingly, we confirmed that very small embryonic-like CD45^−^/SSEA-1^+^ stem cells described by Wu et al. are also enriched in mRNA for transcription factor (Oct-4, Nanog, and Rexo1) but the expression was lower when compared with purified CD45^−^/Lin^−^/CD106^+^ rVSELs isolated in our study. Wu et al. described nonhematopoietic CD45^−^/SSEA-1^+^ cells, which were characterized by small size (4-5 *μ*m), large nucleus, small amount of cytoplasm, expression of PSC transcription factors, and ability to differentiate into the cells from 3-germ layer under special conditions [33]. Thus, these observations may strongly suggest that rat CD45^−^/SSEA-1^+^ and CD45^−^/Lin^−^/CD106^+^ may overlap that would need to be further investigated.

## 5. Conclusions

In conclusion, we identified and characterized in adult rat bone marrow a population of very small stem cells expressing Oct-4A and Nanog markers that shares phenotypic features of previously identified VSELs. The newly identified fraction of rat VSELs (FSC^low^/SSC^low^/CD45^−^/Lin^−^/CD106^+^) may in fact correspond to murine and human VSELs based on their morphology and expression of pluripotent markers such as Oct-4, Nanog, and Rexo1 on mRNA level and protein levels. Comparison of human and murine VSELs as well as newly identified rVSELs is shown in Table 3.

Although our results indicated CD106 as a first positive selection marker for Oct-4A-expressing rat VSELs, further antigens need to be found to enrich these unique stem cells during isolation procedures, when used in coexpression with CD106. Thus, further efforts are needed to exactly define multiantigenic profile of BM-derived rVSELs as well as to further characterize biological properties and functionality of these newly identified rat stem cells.

However, this is the first study optimizing flow cytometric sorting strategies for purification of rat BM-derived Oct-4A^+^ VSELs from both wild type (WT) and eGFP-expressing animals for further experimental purposes in this species. The described phenotype may be employed for further studies of rVSELs in multiple other rat tissues and organs including ovaries and testes. Importantly, the donor eGFP-expressing rVSELs may be followed after transplantation which would allow for further studies of biological properties of these cells* in vivo*.

Importantly, this study opens new perspectives for studying VSELs in several rat tissues and organs in normal healthy conditions as well as for examining their potential regenerative capacity in several unique rat tissue injury models. This would provide a new impact on current knowledge about VSELs including potential regenerative capacity of these unique SCs, which still need to be investigated in* in vitro* as well as* in vivo* studies.

## Figures and Tables

**Table 1:** Antibodies employed in staining for identification and sorting of rat BM-derived populations including rVSELs.

Antibody (anti-rat antigen)	Clone	Fluorochrome	Source
Anti-CD45	OX-1	PE-Cy7/Alx647	BioLegend

Anti-*αβ*-TCR	R73	FITC Alx 647	BD Biosciences BioLegend

Anti-CD2	OX-34	FITC	BD Bioscience

Anti-CD3	1F4	FITC Alx 647	BD Bioscience BioLegend

Anti-CD11b/c	OX-42	FITC Alx 647	BD Bioscience BioLegend

Anti-CD45RA	OX-33	FITC APC	BD Bioscience BioLegend

Anti-CD54	1A29	PE	BD Bioscience

Anti-CD106	MR-106 eBioMR106	PE Biotin	BioLegend eBioscience

Streptavidin	—	PerCp-Cy5.5	BD Bioscience

Anti-SSEA-1	MC-480	PE	BioLegend

**Table 2:** Quantitative real time RT-PCR analysis of mRNA expression for genes related to pluripotency in purified populations of rat BM-derived populations.

Cells fractions	Pluripotent stem cells markers		
	Oct-4	Nanog	Rexo1
	Mean ± SD	Mean ± SD	Mean ± SD
Unfractionated TNCs (control)	1.00	1.00	1.00
CD45^−^/Lin^−^/SSEA-1^+^	4.58 ± 0.91	15.08 ± 6.67	7.90 ± 3.11
CD45^−^/Lin^−^/CD106^+^	34.30 ± 17.35	103.89 ± 27.56	9.34 ± 3.51
CD45^+^/Lin^−^/CD106^+^	2.16 ± 0.91	8.63 ± 2.88	4.90 ± 2.00
CD45^−^/Lin^−^/CD106^+^/CD54^+^	3.11 ± 0.31	13.07 ± 2.72	8.15 ± 2.66
CD45^+^/Lin^−^/CD106^+^/CD54^+^	8.54 ± 3.85	6.69 ± 0.99	4.87 ± 1.73

Results are presented as values of ΔΔCt (average data based on 3 independent experiments; mean ± SD) when compared with whole unfractionated BM tissue (control; computed as 1.00).

**Table 3:** Comparison of major features and phenotype of human and murine VSELs with rat BM-derived VSELs.

Feature	Human VSELs [3, 4, 12, 46]	Murine VSEL [1, 5, 12, 15, 23–25]	Rat VSELs
*Morphology of “very small SCs”* Cells smaller than red blood cells in researched typical species contain large nuclei surrounded by a narrow rim of cytoplasm	+	+	+

Size (by ISS)	6.75 ± 1.04 *µ*m	3.63 ± 0.27 *µ*m	5.32 ± 0.39 *µ*m

Frequency (% of TNCs)	0.01%	0.02%	0.03%

Phenotype currently used for purification (FACS)^*∗*^	CD45^−^/Lin^−^/CD133/1^+^ CD45^−^/Lin^−^/CD34^+^	CD45^−^/Lin^−^/Sca-1^+^	CD45^−^/Lin^−^/CD106^+^

Other surface antigen expression	CD133/1^+^ (AC133^+^), CD34^+^, SSEA-4^+^, AP^+^, c-Met^+^, LIF-R^+^, CXCR4^+^, CD45^−^, Lin^−^	Sca-1^+^, SSEA-1^+^, AP^+^, c-Met^+^, LIF-R^+^, CXCR4^+^, CD45^−^, Lin^−^, HLA-DR^−^, MHC I^−^ CD90^−^, CD29^−^, CD105^−^	CD106^+^, CD54^+/−^, SSEA-1^+^, CD45^−^, Lin^−^

Expression of pluripotent SC transcription factors: Oct-4A and Nanog	+	+	+

(*∗*) Lin – abbreviation indicating major markers characterizing main fractions of hematopoietic cell lineages present in BM tissue such as erythrocytes, monocytes, granulocytes, and subfractions of lymphocytes.

## References

[1] Kucia M., Wysoczynski M., Ratajczak J., Ratajczak M. Z. (2008). Identification of very small embryonic like (VSEL) stem cells in bone marrow. *Cell and Tissue Research*.

[2] Wojakowski W., Tendera M., Kucia M. (2009). Mobilization of bone marrow-derived Oct-4+ SSEA-4+ very small embryonic-like stem cells in patients with acute myocardial infarction. *Journal of the American College of Cardiology*.

[3] Zuba-Surma E. K., Klich I., Greco N. (2010). Optimization of isolation and further characterization of umbilical cord blood-derived very small embryonic/ epiblast-like stem cells (VSELs). *European Journal of Haematology*.

[4] Zuba-Surma E. K., Ratajczak M. Z. (2010). Overview of very small embryonic-like stem cells (VSELs) and methodology of their identification and isolation by flow cytometric methods. *Current Protocols in Cytometry*.

[5] Zuba-Surma E. K., Kucia M., Rui L. (2009). Fetal liver very small embryonic/epiblast like stem cells follow developmental migratory pathway of hematopoietic stem cells. *Annals of the New York Academy of Sciences*.

[6] Kögler G., Sensken S., Airey J. A. (2004). A new human somatic stem cell from placental cord blood with intrinsic pluripotent differentiation potential. *Journal of Experimental Medicine*.

[7] Wakao S., Kitada M., Kuroda Y. (2011). Multilineage-differentiating stress-enduring (Muse) cells are a primary source of induced pluripotent stem cells in human fibroblasts. *Proceedings of the National Academy of Sciences of the United States of America*.

[8] Kuroda Y., Wakao S., Kitada M., Murakami T., Nojima M., Dezawa M. (2013). Isolation, culture and evaluation of multilineage-differentiating stress-enduring (Muse) cells. *Nature Protocols*.

[9] D'Ippolito G., Diabira S., Howard G. A., Menei P., Roos B. A., Schiller P. C. (2004). Marrow-isolated adult multilineage inducible (MIAMI) cells, a unique population of postnatal young and old human cells with extensive expansion and differentiation potential. *Journal of Cell Science*.

[10] Jiang Y., Jahagirdar B. N., Reinhardt R. L. (2002). Pluripotency of mesenchymal stem cells derived from adult marrow. *Nature*.

[11] Beltrami A. P., Cesselli D., Bergamin N. (2007). Multipotent cells can be generated in vitro from several adult human organs (heart, liver, and bone marrow). *Blood*.

[12] Wojakowski W., Kucia M., Zuba-Surma E. (2011). Very small embryonic-like stem cells in cardiovascular repair. *Pharmacology and Therapeutics*.

[13] Ratajczak M. Z., Zuba-Surma E. K., MacHalinski B., Ratajczak J., Kucia M. (2008). Very small embryonic-like (VSEL) stem cells: purification from adult organs, characterization, and biological significance. *Stem Cell Reviews*.

[14] Ratajczak M. Z., Zuba-Surma E., Wojakowski W. (2014). Very small embryonic-like stem cells (VSELs) represent a real challenge in stem cell biology: recent pros and cons in the midst of a lively debate. *Leukemia*.

[15] Zuba-Surma E. K., Kucia M., Ratajczak J., Ratajczak M. Z. (2009). ‘Small stem cells’ in adult tissues: very small embryonic-like stem cells stand up!. *Cytometry Part A*.

[16] Havens A. M., Sun H., Shiozawa Y. (2014). Human and murine very small embryonic-like cells represent multipotent tissue progenitors, in vitro and in vivo. *Stem Cells and Development*.

[17] Sovalat H., Scrofani M., Eidenschenk A., Pasquet S., Rimelen V., Hénon P. (2011). Identification and isolation from either adult human bone marrow or G-CSF-mobilized peripheral blood of CD34^+^/CD133^+^/CXCR4^+^/Lin-CD45^−^ cells, featuring morphological, molecular, and phenotypic characteristics of very small embryonic-l. *Experimental Hematology*.

[18] Virant-Klun I., Skutella T., Hren M. (2013). Isolation of small ssea-4-positive putative stem cells from the ovarian surface epithelium of adult human ovaries by two different methods. *BioMed Research International*.

[19] Virant-Klun I., Skutella T., Kubista M., Vogler A., Sinkovec J., Meden-Vrtovec H. (2013). Expression of pluripotency and oocyte-related genes in single putative stem cells from human adult ovarian surface epithelium cultured in vitro in the presence of follicular fluid. *BioMed Research International*.

[20] Sriraman K., Bhartiya D., Anand S., Bhutda S. (2015). Mouse ovarian very small embryonic-like stem cells resist chemotherapy and retain ability to initiate oocyte-specific differentiation. *Reproductive Sciences*.

[21] Anand S., Patel H., Bhartiya D. (2015). Chemoablated mouse seminiferous tubular cells enriched for very small embryonic-like stem cells undergo spontaneous spermatogenesis in vitro. *Reproductive Biology and Endocrinology*.

[22] Havens A. M., Shiozawa Y., Jung Y. (2013). Human very small embryonic-like cells generate skeletal structures, in vivo. *Stem Cells and Development*.

[23] Kucia M., Reca R., Campbell F. R. (2006). A population of very small embryonic-like (VSEL) CXCR4^+^SSEA-1^+^Oct-4^+^ stem cells identified in adult bone marrow. *Leukemia*.

[24] Zuba-Surma E. K., Kucia M., Wu W. (2008). Very small embryonic-like stem cells are present in adult murine organs: ImageStream-based morphological analysis and distribution studies. *Cytometry Part A*.

[25] Zuba-Surma E. K., Kucia M., Abdel-Latif A. (2008). Morphological characterization of very small embryonic-like stem cells (VSELs) by ImageStream system analysis. *Journal of Cellular and Molecular Medicine*.

[26] Kucia M., Zuba-Surma E., Wysoczynski M. (2006). Physiological and pathological consequences of identification of very small embryonic like (VSEL) stem cells in adult bone marrow. *Journal of Physiology and Pharmacology*.

[27] Shin D. M., Zuba-Surma E. K., Wu W. (2009). Novel epigenetic mechanisms that control pluripotency and quiescence of adult bone marrow-derived Oct4^+^ very small embryonic-like stem cells. *Leukemia*.

[28] Bhartiya D., Mundekar A., Mahale V., Patel H. (2014). Very small embryonic-like stem cells are involved in regeneration of mouse pancreas post-pancreatectomy. *Stem Cell Research and Therapy*.

[29] Kassmer S. H., Jin H., Zhang P.-X. (2013). Very small embryonic-like stem cells from the murine bone marrow differentiate into epithelial cells of the lung. *Stem Cells*.

[30] Iwaki R., Nakatsuka R., Matsuoka Y. M. (2012). Development of a highly efficient method for isolating very small embryonic-like stem cells identified in adult mouse bone and their stem cell characteristics. *ASH Annual Meeting Abstracts*.

[31] Kucia M., Zuba-Surma E. K., Wysoczynski M. (2007). Adult marrow-derived very small embryonic-like stem cells and tissue engineering. *Expert Opinion on Biological Therapy*.

[32] Ratajczak M. Z., Shin D.-M., Liu R. (2012). Very Small Embryonic/Epiblast-Like stem cells (VSELs) and their potential role in aging and organ rejuvenation—an update and comparison to other primitive small stem cells isolated from adult tissues. *Aging*.

[33] Wu J.-H., Wang H.-J., Tan Y.-Z., Li Z.-H. (2012). Characterization of rat very small embryonic-like stem cells and cardiac repair after cell transplantation for myocardial infarction. *Stem Cells and Development*.

[34] Kucia M., Reca R., Jala V. R., Dawn B., Ratajczak J., Ratajczak M. Z. (2005). Bone marrow as a home of heterogenous populations of nonhematopoietic stem cells. *Leukemia*.

[35] Orkin S. H., Zon L. I. (2002). Hematopoiesis and stem cells: plasticity versus developmental heterogeneity. *Nature Immunology*.

[36] Anjos-Afonso F., Bonnet D. (2007). Nonhematopoietic/endothelial SSEA-1^+^ cells define the most primitive progenitors in the adult murine bone marrow mesenchymal compartment. *Blood*.

[37] Springer T. A. (1990). Adhesion receptors of the immune system. *Nature*.

[38] Arkin S., Ferrone S., Lipton J. M. (1995). Modulation of adhesion molecule CD54 expression on CD34^+^ hematopoietic progenitors. *Experimental Hematology*.

[39] Arkin S., Naprstek B., Guarini L., Ferrone S., Lipton J. M. (1991). Expression of intercellular adhesion molecule-1 (CD54) on hematopoietic progenitors. *Blood*.

[40] Koni P. A., Joshi S. K., Temann U.-A., Olson D., Burkly L., Flavell R. A. (2001). Conditional vascular cell adhesion molecule 1 deletion in mice: impaired lymphocyte migration to bone marrow. *Journal of Experimental Medicine*.

[41] Kinashi T., Pierre Y. S., Springer T. A. (1995). Expression of glycophosphatidylinositol-anchored and -non-anchored isoforms of vascular cell adhesion molecule 1 in murine stromal and endothelial cells. *Journal of Leukocyte Biology*.

[42] Perdomo-Arciniegas A.-M., Vernot J.-P. (2011). Co-culture of hematopoietic stem cells with mesenchymal stem cells increases VCAM-1-dependent migration of primitive hematopoietic stem cells. *International Journal of Hematology*.

[43] Basiji D. A., Ortyn W. E., Liang L., Venkatachalam V., Morrissey P. (2007). Cellular image analysis and imaging by flow cytometry. *Clinics in Laboratory Medicine*.

[44] George T. C., Basiji D. A., Hall B. E. (2004). Distinguishing modes of cell death using the ImageStream multispectral imaging flow cytometer. *Cytometry A*.

[45] Ortyn W. E., Hall B. E., George T. C. (2006). Sensitivity measurement and compensation in spectral imaging. *Cytometry Part: A*.

[46] Kucia M., Halasa M., Wysoczynski M. (2007). Morphological and molecular characterization of novel population of CXCR4^+^ SSEA-4^+^ Oct-4^+^ very small embryonic-like cells purified from human cord blood: preliminary report. *Leukemia*.

